# MicroRNAs in Cardiovascular Diseases and Forensic Applications: A Systematic Review of Diagnostic and Post-Mortem Implications

**DOI:** 10.3390/ijms27020825

**Published:** 2026-01-14

**Authors:** Matteo Antonio Sacco, Saverio Gualtieri, Maria Cristina Verrina, Fabrizio Cordasco, Maria Daniela Monterossi, Gioele Grimaldi, Helenia Mastrangelo, Giuseppe Mazza, Isabella Aquila

**Affiliations:** 1 Institute of Legal Medicine, Department of Medical and Surgical Sciences, “Magna Graecia” University, 88100 Catanzaro, Italy; matteosacco@unicz.it (M.A.S.); saveriogualtieri@icloud.com (S.G.); mariacristina.verrina@gmail.com (M.C.V.); cordasco@unicz.it (F.C.); mariadaniela.monterossi@studenti.unicz.it (M.D.M.); gioele.grimaldi@studenti.unicz.it (G.G.); heleniamastrangelo@gmail.com (H.M.); 2 Anesthesia and Intensive Care, Department of Medical and Surgical Sciences, “Magna Graecia” University, 88100 Catanzaro, Italy; giuseppe.mazza@unicz.it

**Keywords:** microRNA, cardiovascular diseases, myocardial infarction, sudden cardiac death, arrhythmogenic cardiomyopathy, post-mortem biomarkers, forensic pathology, autopsy, molecular diagnostics

## Abstract

MicroRNAs (miRNAs) are small non-coding RNA molecules approximately 20–22 nucleotides in length that regulate gene expression at the post-transcriptional level. By binding to target messenger RNAs (mRNAs), miRNAs inhibit translation or induce degradation, thus influencing a wide array of biological processes including development, inflammation, apoptosis, and tissue remodeling. Owing to their remarkable stability and tissue specificity, miRNAs have emerged as promising biomarkers in both clinical and forensic settings. In recent years, increasing evidence has demonstrated their utility in cardiovascular diseases, where they may serve as diagnostic, prognostic, and therapeutic tools. This systematic review aims to comprehensively summarize the role of miRNAs in cardiovascular pathology, focusing on their diagnostic potential in myocardial infarction, sudden cardiac death (SCD), and cardiomyopathies, and their applicability in post-mortem investigations. Following PRISMA guidelines, we screened PubMed, Scopus, and Web of Science databases for studies up to December 2024. The results highlight several miRNAs—including miR-1, miR-133a, miR-208b, miR-499a, and miR-486-5p—as robust markers for ischemic injury and sudden death, even in degraded or formalin-fixed autopsy samples. The high stability of miRNAs under extreme post-mortem conditions reinforces their potential as molecular tools in forensic pathology. Nevertheless, methodological heterogeneity and limited standardization currently hinder their routine application. Future studies should aim to harmonize analytical protocols and validate diagnostic thresholds across larger, well-characterized cohorts to fully exploit miRNAs as reliable molecular biomarkers in both clinical cardiology and forensic medicine.

## 1. Introduction

MicroRNAs (miRNAs) are a class of small non-coding RNA molecules, typically 20–22 nucleotides in length, that play a fundamental role in the regulation of gene expression at the post-transcriptional level [1,2]. They exert their biological effects by binding to complementary sequences within the 3′ untranslated regions (UTRs) of target messenger RNAs (mRNAs), resulting in translational repression or mRNA degradation. This finely tuned regulatory mechanism affects a broad spectrum of physiological processes, including cellular differentiation, proliferation, apoptosis, metabolism, and tissue homeostasis. Because of their small size, evolutionary conservation, and unique patterns of expression across tissues, miRNAs have been increasingly recognized as critical modulators of gene networks in both health and disease. Importantly, their remarkable stability in various biological matrices—including plasma, serum, cerebrospinal fluid, saliva, and even formalin-fixed paraffin-embedded (FFPE) tissue—renders them highly attractive as molecular biomarkers in translational medicine.

In recent years, the field of miRNA research has expanded exponentially, intersecting multiple domains such as oncology, neurology, cardiology, and immunology. In cardiovascular medicine, miRNAs have emerged as key regulators of cardiac physiology and pathophysiology, influencing processes such as cardiac development, response to ischemic stress, electrical conduction, fibrosis, and remodeling [3,4,5]. Dysregulation of specific cardiac miRNAs has been consistently associated with acute myocardial infarction (AMI), heart failure, cardiomyopathies, and sudden cardiac death (SCD). For instance, miR-1 and miR-133a are muscle-enriched miRNAs known to regulate cardiomyocyte differentiation and electrical conduction; miR-208b and miR-499a are highly specific for cardiac tissue and are released into circulation during myocardial necrosis. These molecules not only serve as molecular fingerprints of cardiac injury but also provide mechanistic insight into the cellular responses underlying disease progression. Their presence in body fluids and tissues even after death suggests that they could represent a bridge between clinical cardiology and forensic pathology, improving post-mortem diagnostic accuracy when conventional morphological indicators are ambiguous or absent.

MicroRNAs have been extensively investigated in cardiovascular research, where they regulate essential processes such as cardiomyocyte differentiation, electrical conduction, hypertrophy, ischemia–reperfusion injury, and fibrotic remodeling. Several landmark studies have demonstrated that cardiac-enriched microRNAs—including miR-1, miR-133a/b, miR-208a/b, and miR-499—constitute highly specific molecular signatures of myocardial injury and heart failure, providing mechanistic and diagnostic insight into acute and chronic cardiac pathology [3,6,7,8]. Recent reviews have also emphasized their role as dynamic biomarkers measurable in blood, serum, exosomes, and formalin-fixed tissues, thus enabling precise molecular phenotyping of cardiac conditions, including acute myocardial infarction (AMI), arrhythmogenic cardiomyopathy, myocarditis, and stress cardiomyopathy [4,9,10].

Beyond clinical cardiology, an emerging body of literature indicates that microRNAs maintain exceptional post-mortem stability, thereby offering unprecedented opportunities in forensic medicine. Unlike conventional mRNA or protein markers—which undergo rapid autolytic and environmental degradation—microRNAs demonstrate resilience to temperature fluctuations, prolonged post-mortem intervals, decomposition, and formalin fixation, remaining quantifiable even in severely compromised tissues [1,2,11]. This biochemical robustness has led to increasing interest in their application for determining cause of death, identifying early myocardial ischemia, differentiating cardiac from non-cardiac etiologies of collapse, and supporting the “molecular autopsy” in cases of unexplained sudden death. Studies on post-mortem blood, pericardial fluid, myocardium, and FFPE blocks have consistently shown that cardiac microRNAs can discriminate AMI from other fatal conditions, provide molecular evidence of silent ischemia, and reveal arrhythmogenic processes in structurally unremarkable hearts [5,12,13,14].

Despite the abundance of reviews discussing microRNAs in cardiovascular disease, very few have specifically addressed their expression in human cardiac tissue and post-mortem samples, and none have systematically integrated clinical and forensic applications into a unified diagnostic framework. This represents a significant gap, given that molecular markers capable of identifying subtle myocardial injury or arrhythmic triggers are urgently needed in forensic pathology, where morphological findings are often inconclusive. For this reason, the present systematic review focuses exclusively on microRNA expression in cardiac tissue and post-mortem specimens, aiming to clarify their diagnostic value, analytical challenges, and potential future incorporation into standardized medico-legal protocols.

The application of miRNAs in forensic medicine represents a rapidly expanding frontier. The post-mortem interval (PMI), environmental conditions, and tissue decomposition have traditionally limited the reliability of molecular analyses in death investigations. However, numerous studies have demonstrated that miRNAs remain detectable and quantifiable in autopsy specimens long after death, due to their intrinsic resistance to enzymatic degradation and chemical stress. This molecular resilience allows their use not only for tissue identification but also for cause-of-death determination, particularly in cases of unexplained or sudden deaths where histological findings are inconclusive. For example, miRNAs can distinguish between cardiac and non-cardiac causes of death, identify ischemic or inflammatory lesions, and potentially contribute to molecular estimation of the PMI [6,7]

Furthermore, the pathophysiological relevance of miRNAs extends beyond their diagnostic capacity. Several experimental studies have revealed that altered miRNA expression contributes to cardiac fibrosis, hypertrophy, apoptosis, and electrical instability—processes that underlie arrhythmogenesis and structural heart disease [8]. MiRNAs such as miR-21, miR-34a, and miR-29b have been implicated in fibrotic remodeling and myocardial scarring, while miR-15 family members regulate ischemia-induced apoptosis. This expanding molecular landscape provides not only diagnostic markers but also potential therapeutic targets for preventing adverse cardiac remodeling. The dual role of miRNAs—as mechanistic regulators and measurable biomarkers—underscores their growing importance in both precision cardiology and forensic pathology [12,15]

From a forensic standpoint, the ability to detect cardiac-specific miRNAs in post-mortem tissues opens new perspectives in the molecular investigation of sudden deaths. Traditional histopathological criteria often fail to distinguish subtle or early myocardial ischemic changes, especially when death occurs rapidly and autolysis obscures cellular detail. In such contexts, miRNA profiling may provide molecular evidence of cardiac involvement even when macroscopic and microscopic features appear unremarkable. The identification of specific miRNA signatures associated with acute myocardial infarction, arrhythmogenic cardiomyopathy, or stress-induced cardiomyopathy (Takotsubo syndrome) could revolutionize the diagnostic paradigm of forensic autopsies, offering an objective and reproducible molecular approach to complement classical pathology [13].

Nevertheless, despite these promising findings, several challenges remain unresolved. The lack of standardized analytical protocols, variability in RNA extraction and normalization methods, and differences in the selection of reference genes have limited cross-study comparability. Moreover, the interpretation of miRNA expression in post-mortem samples must account for potential confounding factors such as PMI, environmental exposure, and tissue degradation kinetics. These issues underscore the necessity of harmonized methodologies and large-scale validation studies to ensure reproducibility and forensic applicability.

In light of these considerations, the present systematic review aims to provide a comprehensive synthesis of the available literature on the role of miRNAs in cardiovascular diseases with a specific focus on their application in forensic contexts. By integrating findings from both clinical and post-mortem studies, this review seeks to delineate the diagnostic and mechanistic significance of cardiac miRNAs, highlight their potential as molecular tools for determining the cause of death, and discuss future directions for their implementation in routine medico-legal practice.

## 2. Materials and Methods

This systematic review was conducted according to the Preferred Reporting Items for Systematic Reviews and Meta-Analyses (PRISMA) guidelines, which provide a rigorous and transparent framework for identifying, screening, and synthesizing scientific evidence. The aim was to gather and critically evaluate all available studies investigating the role of microRNAs (miRNAs) in cardiovascular diseases, with particular focus on their diagnostic and forensic applications in post-mortem settings. The protocol was designed to ensure methodological reproducibility and to minimize selection bias, adhering to international standards for systematic reviews in molecular medicine.

### 2.1. Literature Search Strategy

A comprehensive literature search was performed across three major electronic databases—PubMed, Scopus, and Web of Science (WoS)—to identify studies published up to December 2024. No time restrictions were initially applied, but filters were later used to refine the selection to peer-reviewed original research articles written in English. The search strategy included combinations of controlled vocabulary terms and free-text keywords such as “microRNA” OR “miRNA” AND “cardiac” OR “myocardial infarction” OR “sudden cardiac death” OR “autopsy” OR “forensic”. Boolean operators were used to maximize sensitivity and ensure comprehensive retrieval of relevant studies. Reference lists of included articles and key reviews were also manually screened to identify additional eligible works not captured by database indexing.

**Figure 1:**
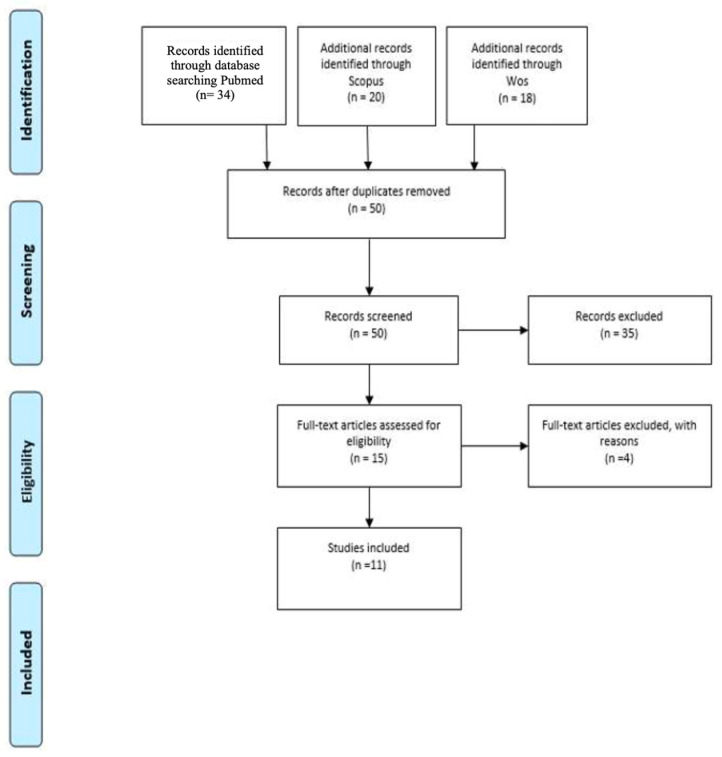
PRISMA Flowchart.

All retrieved references were imported into a bibliographic management software, and duplicate entries were automatically removed. The search process was documented in detail to ensure transparency, and the overall workflow was summarized in a PRISMA flow diagram (Figure 1), outlining the number of records identified, screened, excluded, and ultimately included in the review.

### 2.2. Inclusion and Exclusion Criteria

Studies were considered eligible if they met the following criteria:

(1) Original human studies analyzing miRNA expression in cardiac tissue or post-mortem samples;
(2) Research addressing the pathophysiological role or diagnostic utility of miRNAs in cardiovascular diseases, including myocardial infarction, cardiomyopathies, and sudden cardiac death;
(3) Use of validated quantitative molecular techniques, such as reverse transcription quantitative polymerase chain reaction (RT-qPCR), next-generation sequencing (NGS), microarray analysis, or bioinformatic pipelines;
(4) Provision of quantitative or semi-quantitative data linking miRNA expression levels to specific pathological findings or forensic interpretations.

Exclusion criteria included:

(a) non-English language publications;
(b) reviews, meta-analyses, editorials, or conference abstracts without original data;
(c) studies involving only animal or in vitro models;
(d) incomplete datasets or insufficient methodological details preventing data extraction;
(e) absence of clear association between miRNA expression and cardiac or forensic outcomes.

When studies presented overlapping data (e.g., same patient cohort or dataset analyzed in multiple papers), only the most comprehensive and recent publication was included to avoid duplication of information.

### 2.3. Study Selection Process

The selection process was carried out independently by two reviewers with expertise in forensic pathology and molecular biology. Titles and abstracts of all identified studies were screened to exclude irrelevant works. Potentially eligible studies were then subjected to full-text assessment. Any disagreement between reviewers was resolved through discussion or, when necessary, by consulting a third senior reviewer to reach consensus. The inter-reviewer agreement was high, reflecting the clarity of the inclusion criteria and the objectivity of the selection process.

Each included study was then coded with a unique identifier, and its bibliographic data were cross-checked for accuracy. The final dataset represented a balanced collection of both clinical and post-mortem investigations, ensuring a comprehensive overview of miRNA behavior across different experimental and pathological contexts.

### 2.4. Data Extraction and Synthesis

Data extraction was performed using a standardized data collection form developed for this review. The following information was recorded for each included article: title, authors, year of publication, country, study design, number and type of samples analyzed (e.g., cardiac tissue, serum, plasma, pericardial fluid, or FFPE blocks), analytical methods employed, specific miRNAs investigated, principal findings, and reported forensic or diagnostic implications. Where available, quantitative metrics such as fold-change expression, sensitivity, specificity, and area under the receiver operating characteristic curve (AUC) were documented.

The extracted data were then verified for consistency by a second reviewer. Discrepancies or ambiguities were resolved by consulting the original publication. Given the heterogeneity of study designs and outcome measures, a narrative synthesis approach was adopted rather than a formal meta-analysis. Studies were grouped thematically according to disease type (e.g., myocardial infarction, sudden cardiac death, arrhythmogenic cardiomyopathy) and methodological approach (e.g., tissue-based, circulating, or exosomal miRNAs).

Whenever feasible, data were summarized in comparative tables to facilitate visualization of trends across studies. These tables highlight the diagnostic potential and forensic relevance of specific miRNAs, allowing direct comparison between molecular signatures identified in different experimental settings.

### 2.5. Quality Assessment

To ensure robustness and reliability, a qualitative evaluation of methodological quality was performed for each included study. Criteria considered included sample size, description of post-mortem conditions, RNA extraction and quantification methods, normalization strategies, statistical analysis, and reporting transparency. Studies with incomplete methodological details or lacking appropriate controls were interpreted with caution in the synthesis of results. Although a formal risk-of-bias tool was not applied due to the descriptive nature of many included works, methodological rigor and reproducibility were key factors influencing data interpretation and strength of conclusions (Table 1).

## 3. Results

A total of eleven studies met the inclusion criteria and were analyzed in detail. Collectively, these works explored the diagnostic, prognostic, and mechanistic significance of microRNAs (miRNAs) in cardiovascular diseases, with a particular focus on their application to forensic pathology and post-mortem diagnosis. The selected studies covered both clinical and autopsy-based designs, employing various analytical platforms including RT-qPCR, next-generation sequencing (NGS), and RNA sequencing. Biological matrices ranged from fresh cardiac tissue and formalin-fixed paraffin-embedded (FFPE) samples to blood, serum, and pericardial fluid, offering a comprehensive overview of miRNA behavior under different preservation and post-mortem conditions. This diversity of study designs provided important insights into the robustness and diagnostic consistency of miRNA expression profiles across multiple experimental settings.

Several studies consistently demonstrated that certain miRNAs, particularly miR-1, miR-133a/b, miR-208b, and miR-499a, are deregulated in acute myocardial infarction (AMI) and can serve as reliable molecular markers of ischemic injury. Bostjancic et al. (2010) [9] performed one of the earliest post-mortem analyses of cardiac miRNAs, showing that miR-208 was markedly upregulated in infarcted myocardial tissue, particularly in acute cases (<24 h after death), while miR-1 and miR-133a/b were downregulated. The downregulation of these latter miRNAs mirrored the expression patterns observed in fetal cardiac tissue, suggesting a process of genetic reprogramming and cardiomyocyte dedifferentiation following ischemic injury. These results supported the concept that miR-208 is a specific molecular indicator of acute myocardial infarction and can distinguish infarcted myocardium from normal cardiac tissue even after death. Menathung et al. (2017) [12] further validated this diagnostic potential by demonstrating that miR-499 was significantly elevated in post-mortem blood from individuals who died of AMI, while miR-133a and miR-208b exhibited increasing trends. Notably, miR-499 maintained stability for extended post-mortem intervals, underscoring its potential as a circulating biomarker for the forensic identification of infarction in cases where histological evidence is equivocal or absent. Similarly, Kakimoto et al. (2015) [10] examined FFPE autopsy samples and confirmed that miR-208b and miR-499a remain detectable and quantifiable despite prolonged fixation. The study revealed a downregulation of miR-1 in infarcted tissues and a variable expression of miR-208b and miR-499a depending on the stage of infarction, suggesting that temporal expression profiles of miRNAs could assist in determining the evolution and timing of ischemic events. This finding was particularly significant for forensic contexts, as it demonstrates that reliable molecular information can be obtained even from archived autopsy material. The work of Wilmes et al. (2024) [11] contributed additional nuance by analyzing miR-21, miR-939, and miR-30e expression in post-mortem myocardial tissue. They found that miR-21 and miR-939 were consistently upregulated in infarcted areas, whereas miR-30e was more abundant in non-infarcted zones. This differential expression pattern offers a molecular means of distinguishing ischemic from non-ischemic myocardium, a task that can be challenging using conventional histopathology alone, especially in decomposed or structurally preserved hearts. Together, these studies emphasize that cardiac-specific miRNAs can serve as precise indicators of myocardial injury and can remain intact long enough to retain diagnostic value in forensic examinations.

The role of miRNAs in modulating immune and inflammatory responses following myocardial infarction has also been highlighted in the literature. Zidar et al. (2011) [16] investigated the expression of miR-146a, miR-150, and miR-155 in infarcted human cardiac tissue and found significant alterations in their expression among patients who experienced ventricular rupture, a fatal complication of excessive inflammatory activity. Specifically, miR-146a was upregulated, while miR-150 was markedly reduced, suggesting a protective role of miR-150 in modulating inflammatory cascades. MiR-155 showed variable expression, consistent with its function in metalloproteinase regulation and leukocyte recruitment. These findings illustrate how miRNAs may act as molecular intermediaries linking immune activation to structural cardiac failure, providing new mechanistic insight into post-infarction complications that can lead to death.

Beyond solid tissue, several studies have investigated the diagnostic performance of circulating and exosomal miRNAs in post-mortem fluids. Kim et al. (2024) [20] conducted a large-scale analysis of miRNAs in serum and pericardial fluid obtained from patients with ischemic heart disease. Using NGS and RT-qPCR, they identified miR-486-5p as a robust biomarker, highly overexpressed in cases of acute myocardial infarction, particularly in association with advanced atherosclerotic plaques. The detection of miR-486-5p and other exosomal miRNAs in pericardial fluid not only reflects cardiac pathology but also provides a powerful non-invasive molecular tool for forensic diagnosis when heart tissue is unavailable or decomposed. The ability to assess cardiac molecular alterations through fluid-based biomarkers represents a major advancement in post-mortem molecular diagnostics.

A number of studies have also addressed the use of miRNAs in sudden cardiac death (SCD), a condition of major forensic importance often lacking clear structural correlates. Yan et al. (2021) [14] analyzed the expression of miR-3113-5p, miR-223-3p, miR-133a-3p, and miR-499a-5p in cardiac tissues from both structurally positive and negative SCD cases. All four miRNAs were significantly elevated compared to controls, with higher expression in autopsy-negative cases, suggesting their potential in identifying arrhythmic or electrical deaths where traditional morphology fails to provide answers. The combination of two or more miRNAs enhanced diagnostic accuracy, supporting the development of molecular panels tailored to forensic application. Li et al. (2022) [17] further refined this approach by demonstrating that miR-126-5p and miR-499a-5p could differentiate coronary artery disease–related SCD from other causes of sudden death, even in the absence of macroscopic or histological signs of acute ischemia. Bernini Di Michele et al. (2023) [18] corroborated these findings by reporting that miR-208b and miR-499a-5p were consistently overexpressed in cardiac tissues of SCD victims, whereas miR-1 and miR-133a were associated with cases showing structural cardiac abnormalities. The authors also highlighted the stability of these markers in serum and plasma, confirming their resilience and diagnostic value over extended post-mortem intervals.

An additional contribution of particular relevance is the study by Mildeberger et al. [15], which quantitatively compared the expression of miR-1, miR-133a, and miR-26a in both cardiac tissue and whole blood from post-mortem cases of myocardial infarction. The authors demonstrated a consistent upregulation of miR-1 and miR-133a in infarcted myocardium, accompanied by a marked reduction in miR-26a, a regulator of ionic homeostasis and cardiac excitability. Importantly, the study also evaluated these microRNAs in whole blood, showing that their differential expression profiles remain detectable and diagnostically informative after death. ROC curve analysis confirmed high discriminative accuracy for miR-1 and miR-133a in distinguishing infarcted from non-infarcted hearts, supporting their potential use as molecular biomarkers when histopathology is equivocal or compromised by autolysis. This work represents one of the most robust demonstrations of cross-matrix reproducibility in forensic microRNA analysis, reinforcing the stability and diagnostic value of cardiac-specific microRNAs across different post-mortem biological substrates.

Finally, the study by Bonet et al. (2024) [19] expanded the understanding of miRNAs in structural heart diseases, focusing on arrhythmogenic cardiomyopathy (ACM), a genetic disorder frequently implicated in sudden cardiac death among young individuals. Through transcriptomic analysis of autopsy-derived cardiac samples, the authors identified nearly seven hundred genes and eight miRNAs with differential expression profiles. Upregulated miRNAs such as miR-135a-5p, miR-140-3p, and miR-145-5p were linked to inflammatory and fibrotic signaling pathways, while downregulated miRNAs including miR-125a-5p and miR-486-5p were associated with mitochondrial and oxidative stress regulation. These findings indicate that miRNAs contribute to the molecular pathogenesis of ACM and could serve as potential biomarkers for distinguishing this condition from other causes of sudden death. Their identification also opens new perspectives for targeted molecular therapies aimed at modulating the miRNA–mRNA interactome in inherited cardiomyopathies.

Across all studies, a coherent pattern emerges: cardiac miRNAs display disease-specific expression profiles that remain quantifiable and diagnostically informative even in degraded or preserved post-mortem samples. The simultaneous analysis of tissue, serum, and exosomal miRNAs enhances the reliability of molecular diagnosis and provides complementary evidence to histopathology and toxicology. Nevertheless, methodological heterogeneity among studies—including differences in sample handling, RNA extraction, normalization, and post-mortem interval—continues to limit comparability and reproducibility. Despite these challenges, the cumulative evidence strongly supports miRNAs as stable, disease-specific, and forensically robust biomarkers capable of bridging the gap between molecular cardiology and forensic pathology.

## 4. Discussion

The findings synthesized in this review collectively confirm that microRNAs play a pivotal role in cardiovascular pathophysiology and represent promising molecular tools for both clinical diagnostics and forensic investigations. Their short sequence length, evolutionary conservation, and high tissue specificity confer remarkable analytical advantages, particularly in cardiac tissues where conventional biomarkers are often limited by instability or degradation [21]. In the context of cardiovascular pathology, miRNAs act as regulators of multiple cellular processes including cardiomyocyte apoptosis, necrosis, hypertrophy, inflammation, and remodeling, all of which are key determinants of disease progression and outcome. Their deregulated expression reflects both the underlying pathophysiological mechanisms and the cellular response to injury, thus enabling the identification of molecular fingerprints that correspond to specific cardiac events such as acute myocardial infarction, ventricular rupture, or sudden cardiac death [22].

A consistent theme emerging from the reviewed studies is the diagnostic and prognostic significance of a core set of cardiac-enriched miRNAs—namely miR-1, miR-133a/b, miR-208b, miR-499a, and miR-486-5p. These molecules exhibit strong cardiac specificity, high abundance in myocardial tissue, and quantifiable release into circulation following cardiomyocyte damage. Their expression profiles remain detectable not only in fresh tissue and serum but also in formalin-fixed paraffin-embedded (FFPE) blocks, pericardial fluid, and post-mortem blood, demonstrating an exceptional degree of molecular resilience [23]. This stability is particularly advantageous for forensic purposes, where degradation of RNA and protein markers often precludes molecular analysis. The persistence of miRNAs under adverse conditions such as decomposition, prolonged storage, or chemical fixation has been attributed to their small size, association with RNA-binding proteins, and encapsulation within exosomes or microvesicles that protect them from enzymatic degradation. These features collectively make miRNAs superior to conventional mRNA or protein-based markers for post-mortem diagnostics [24].

The ability of specific miRNAs to discriminate among different causes of cardiac death has profound implications for forensic medicine. In particular, miR-208b and miR-499a have been repeatedly shown to increase sharply in myocardial infarction, providing objective molecular evidence of ischemic injury that can complement or even replace histological confirmation [25]. Their quantification could allow pathologists to identify myocardial infarction in cases where microscopic changes are minimal, ambiguous, or masked by autolysis. Similarly, the combined evaluation of miR-133a and miR-1, which are involved in cardiac conduction and myocyte differentiation, can offer information about electrical and arrhythmic disturbances contributing to sudden death. The integration of these molecular parameters into the routine forensic workflow could transform the diagnostic paradigm from one based primarily on morphology to a multimodal framework combining structural, biochemical, and genetic evidence.

Beyond ischemic injury, the reviewed studies also demonstrate that miRNAs mediate critical post-infarction processes such as inflammation and tissue repair. The upregulation of miR-146a and miR-155 and the downregulation of miR-150 in infarcted tissue, as reported by Zidar et al., highlight their involvement in the inflammatory response and extracellular matrix remodeling that may lead to ventricular rupture. These findings indicate that miRNAs not only act as diagnostic indicators but also reflect dynamic molecular cascades underlying fatal complications. By quantifying their expression, forensic pathologists may gain insight into the timing, severity, and cellular mechanisms of myocardial injury. Moreover, the association of miRNAs with apoptotic and fibrotic signaling pathways provides a molecular continuum linking acute ischemic events with chronic remodeling processes observed in cardiomyopathies.

A particularly relevant advancement comes from the detection of circulating and exosomal miRNAs in post-mortem fluids such as serum and pericardial effusion [26]. The study by Kim et al. demonstrated that miR-486-5p and other vesicle-associated miRNAs remain quantifiable even after death and correlate with the presence of advanced atherosclerosis and ischemic heart disease. This approach opens new opportunities for forensic analysis in cases where heart tissue is unavailable or severely decomposed. The ability to isolate exosomal miRNAs from small fluid samples not only enhances diagnostic feasibility but also supports retrospective analyses in long-term preserved specimens, where histology and immunohistochemistry may no longer be informative. This represents a significant methodological innovation that could expand the toolkit of molecular autopsy beyond DNA sequencing and conventional toxicology.

The implications of miRNA research extend further into the field of sudden cardiac death, one of the most challenging diagnostic entities in forensic medicine. Traditional autopsies often fail to reveal the cause of death in cases where electrical or functional disturbances precede structural damage. In such scenarios, molecular biomarkers can provide the missing link between clinical presentation and fatal outcome. Studies by Yan et al. and Li et al. have shown that specific miRNA panels can distinguish sudden deaths caused by arrhythmic or coronary mechanisms even when macroscopic and microscopic findings are inconclusive. These observations are of exceptional medico-legal importance, as they provide molecular evidence capable of reclassifying previously unexplained deaths and clarifying the presence of latent cardiac pathology. Moreover, the expression of miRNAs such as miR-133a and miR-499a in autopsy-negative SCD suggests that these molecules can identify subtle electrical or functional disturbances at the molecular level, marking a paradigm shift in how forensic pathologists interpret unexplained cardiac fatalities.

In structural heart diseases such as arrhythmogenic cardiomyopathy, miRNAs further demonstrate their value as mechanistic and diagnostic biomarkers. The transcriptomic analysis conducted by Bonet et al. revealed complex miRNA–mRNA interaction networks that govern mitochondrial respiration, oxidative stress response, and inflammatory pathways. This molecular complexity reinforces the concept that cardiomyopathies are not merely structural disorders but also involve intricate epigenetic and post-transcriptional regulation. From a translational perspective, this insight opens avenues for targeted therapeutic interventions aimed at modulating specific miRNA–mRNA axes, potentially offering new strategies to prevent arrhythmic sudden death in genetically predisposed individuals.

Despite these advances, several limitations must be acknowledged. The heterogeneity of experimental designs, post-mortem intervals, and analytical methods across studies introduces variability that complicates data comparison and interpretation. Differences in RNA extraction protocols, normalization strategies, and choice of reference genes remain significant sources of bias. Furthermore, few studies have systematically addressed the impact of environmental conditions, body temperature, and tissue autolysis on miRNA degradation kinetics. Standardization of pre-analytical and analytical workflows is therefore essential to ensure reproducibility and to facilitate the integration of miRNA profiling into routine forensic practice. The establishment of quantitative thresholds and validated reference ranges for specific miRNAs will be a necessary step toward clinical and legal acceptance of these biomarkers.

Future research should move toward multi-dimensional and integrative approaches that combine miRNA expression profiling with complementary molecular layers such as mRNA, proteomics, and metabolomics. Multi-omic integration can provide a comprehensive systems-level understanding of post-mortem molecular changes and clarify causal relationships between gene regulation and tissue pathology. Additionally, the use of artificial intelligence and machine learning could significantly enhance the interpretation of complex datasets, allowing automated pattern recognition, biomarker prioritization, and predictive modeling of cause of death. By merging molecular, histological, and digital data, AI-driven tools could contribute to a new generation of precision forensic pathology, where objective molecular signatures complement expert interpretation.

In summary, the cumulative evidence supports the view that microRNAs are among the most promising molecular biomarkers for cardiovascular and forensic applications. Their stability, specificity, and mechanistic relevance make them uniquely suited for the investigation of cardiac deaths, especially when morphological evidence is insufficient or ambiguous. As technology advances and standardization improves, miRNA-based diagnostics have the potential to transform both clinical cardiology and forensic pathology, bridging the gap between molecular mechanisms and the ultimate determination of cause of death.

### Critical Appraisal of Included Studies

A critical comparison of the included studies reveals substantial heterogeneity in methodological design, analytical strategies, and post-mortem conditions, which directly affects the reliability and comparability of their findings. Sample sizes ranged from small case–control series of fewer than 10 hearts [9,10,16] to larger molecular cohorts exceeding 40 samples [14,17,18], limiting the statistical power of several investigations and increasing the risk of type-I and type-II errors. Analytical methods were likewise heterogeneous: some studies employed RT-qPCR on fresh myocardium, whereas others used FFPE blocks [10], post-mortem whole blood [12], pericardial fluid [20], or RNA-seq-based discovery platforms [19]. These methodological discrepancies partly explain the variability in reported fold-changes and complicate direct comparison across studies.

Post-mortem conditions—including the post-mortem interval, temperature, tissue preservation, and degree of autolysis—were inconsistently reported and rarely standardized, despite being known determinants of RNA degradation. Only a minority of studies explicitly quantified PMI or assessed RNA integrity, making it difficult to evaluate the extent to which observed miRNA expression changes reflect true biological alterations rather than degradation artifacts. Nevertheless, the consistent upregulation of miR-208b and miR-499a across independent settings, including FFPE tissue and post-mortem blood, strongly supports their robustness as biomarkers of acute myocardial injury. In contrast, miRNAs such as miR-26a, miR-150, or miR-155 showed greater variability, suggesting that they may be more sensitive to pre-analytical factors or tissue-specific pathophysiology.

Overall, while the included studies collectively support the forensic potential of cardiac microRNAs, the lack of methodological standardization underscores the need for harmonized protocols, reference controls, and validation across larger, well-characterized post-mortem cohorts.

From a forensic perspective, cardiac microRNAs offer diagnostic advantages that overcome several limitations of conventional autopsy findings. Early myocardial ischemia frequently lacks macroscopic or microscopic hallmarks, particularly when the agonal period is short or when decomposition obscures cellular morphology. In these scenarios, the detection of cardiac-enriched miRNAs such as miR-208b, miR-499a, or miR-1 may provide molecular confirmation of cardiomyocyte necrosis even when histology is inconclusive. Their stability in degraded tissues, FFPE blocks, post-mortem blood, and pericardial fluid—as demonstrated in multiple studies—suggests that microRNA profiling could become a complementary tool in routine forensic workflows, especially in cases of sudden cardiac death with negative gross findings.

Furthermore, the ability to discriminate ischemic from non-ischemic myocardial regions [11], to identify silent coronary ischemia [17], or to differentiate unexplained sudden death from structurally evident SCD [14], highlights the potential of microRNAs in the molecular autopsy. In practice, these biomarkers could be integrated into sampling protocols together with toxicology, immunohistochemistry, and genetic screening, helping pathologists establish the cause and timing of death with greater accuracy. Future forensic guidelines may incorporate miRNA panels as part of standardized algorithms for evaluating suspected cardiac deaths, particularly in decomposed, exhumed, or histologically ambiguous cases.

## 5. Conclusions

The collective evidence reviewed in this work demonstrates that microRNAs represent a highly stable and biologically informative class of molecular regulators with exceptional diagnostic and forensic potential in cardiovascular diseases. Their ability to persist in tissues and fluids long after death, combined with their tissue specificity and regulatory influence on critical cardiac pathways, positions them as indispensable biomarkers for elucidating the mechanisms underlying myocardial infarction, cardiomyopathies, and sudden cardiac death. Unlike conventional biochemical or histological parameters, miRNAs are not significantly affected by post-mortem degradation, fixation, or storage conditions, allowing reliable molecular assessment even when morphological and immunohistochemical evidence is compromised.

The consistent upregulation of cardiac-enriched miRNAs such as miR-1, miR-133a/b, miR-208b, miR-499a, and miR-486-5p across independent studies confirms their diagnostic robustness and their applicability to both clinical and forensic settings. Their expression profiles provide molecular insight into the temporal evolution and severity of ischemic injury, as well as the inflammatory and apoptotic cascades that accompany fatal cardiac events. In forensic practice, the quantification of these miRNAs could serve as a complementary tool to conventional autopsy, enabling the differentiation between ischemic, arrhythmic, and structural causes of death and improving diagnostic accuracy in cases where the cause of death remains uncertain [22,23,24,25,26,27,28,29,30].

Nonetheless, significant methodological challenges persist. The lack of standardized procedures for RNA extraction, normalization, and data interpretation continues to limit the comparability of results across laboratories. Furthermore, confounding factors such as post-mortem interval, environmental exposure, and interindividual variability require systematic evaluation before miRNA assays can be integrated into medico-legal protocols. The development of harmonized analytical workflows and validated diagnostic thresholds will therefore be essential for translating miRNA research into forensic application.

Looking forward, future investigations should adopt integrative multi-omic frameworks that combine miRNA data with transcriptomic, proteomic, and metabolomic signatures, thus enabling a comprehensive systems-level understanding of cardiac pathology in both living and post-mortem contexts [11,20,22,31,32,33,34,35,36,37]. The application of artificial intelligence and machine learning to these datasets holds promise for the creation of predictive diagnostic models capable of distinguishing subtle molecular patterns of cardiac injury. By bridging molecular cardiology and forensic pathology, miRNA profiling has the potential to redefine cause-of-death investigation, transforming autopsy practice into a truly molecular discipline grounded in quantitative and reproducible evidence.

In conclusion, microRNAs stand at the frontier of precision diagnostics and forensic molecular medicine. Their integration into routine workflows, supported by rigorous methodological standardization and interdisciplinary collaboration, could mark a decisive step toward a new era of molecular autopsy—one in which the silent language of RNA provides objective answers to the most elusive questions of life and death.

## Figures and Tables

**Table 1:** Cardiac microRNAs investigated in clinical and post-mortem studies with methodological and forensic relevance.

Study (Ref.)	Sample Size (n)	Method	Post-Mortem Conditions	miRNAs Investigated	Expression Pattern (↑/↓)	Matrix	Condition/Pathology	**Diagnostic/** **Forensic Value**
Bostjancic et al., 2010 [9]	31	RT-qPCR	Myocardial tissue; no PMI reported	miR-1, miR-133a/b, miR-208	miR-208 ↑; miR-1 ↓; miR-133 ↓	Heart tissue	Acute & chronic MI	miR-208 highly specific for acute MI; ischemic remodeling signature
Zidar et al., 2011 [16]	20	RT-qPCR	Fresh tissue; PMI not specified	miR-146a, miR-150, miR-155	miR-146a ↑; miR-150 ↓; miR-155 variable	Infarcted myocardium	Ventricular rupture post-MI	Indicates inflammatory activation & ECM degradation
Kakimoto et al., 2015 [10]	14	RT-qPCR; electrophoresis	FFPE autopsy samples; long-term fixation	miR-1, miR-208b, miR-499a	miR-208b ↑; miR-499a ↑; miR-1 ↓	FFPE cardiac tissue	Acute MI	Confirms miRNA stability in FFPE; supports retrospective MI diagnosis
Menathung et al., 2017 [12]	24	RT-qPCR	Post-mortem blood ≤ 48 h	miR-133a, miR-208b, miR-499	miR-499 ↑↑; miR-133a ↑; miR-208b ↑	PM whole blood	MI	Strong stability of miR-499 in PM blood; high diagnostic performance
Yan et al., 2021 [14]	45	RT-qPCR	PMI < 24–48 h (reported)	miR-3113-5p, miR-223-3p, miR-133a-3p, miR-499a-5p	All ↑	Cardiac tissue	SCD, autopsy-positive & negative	Higher expression in autopsy-negative SCD; useful for unexplained sudden death
Li et al., 2022 [17]	36	RT-qPCR	Autopsy hearts; PMI not reported	miR-126-5p, miR-134-5p, miR-499a-5p	miR-126 ↓; miR-134 ↓; miR-499 ↓	Cardiac tissue	CAD-related SCD	Differentiates coronary vs. non-coronary SCD even without macroscopic lesions
Mildeberger et al., 2023 [15]	40	RT-qPCR; ROC analysis	Tissue & whole blood; PMI controlled	miR-1, miR-133a, miR-26a	miR-1 ↑; miR-133a ↑; miR-26a ↓	Tissue + PM whole blood	MI	High cross-matrix reproducibility; strong discriminative accuracy
Bernini Di Michele et al., 2023 [18]	52	RT-PCR; quantitative assays	Tissue + fluids; various PMI	miR-1, miR-133a/b, miR-208b, miR-499a-5p	miR-208b ↑; miR-499a ↑; miR-1 ↑; miR-133 ↑	Heart tissue + fluids	SCD	Demonstrates stability and diagnostic potential across PM matrices
Wilmes et al., 2023–24 [11]	27	RT-qPCR	Fresh vs. infarcted areas; autopsy	miR-21, miR-939, miR-30e	miR-21 ↑; miR-939 ↑; miR-30e ↓	Myocardium	MI	Molecular differentiation between ischemic and non-ischemic zones
Bonet et al., 2024 [19]	17	RNA-seq	Autopsy ACM hearts	Multiple miRNAs	miR-135a-5p ↑; miR-140-3p ↑; miR-145-5p ↑; miR-125a-5p ↓; miR-486 ↓	Heart tissue	Arrhythmogenic cardiomyopathy	Identifies ACM-specific miRNA clusters; supports forensic genetic diagnosis
Kim et al., 2024 [20]	60	NGS + RT-qPCR	Serum & pericardial fluid from autopsy	miR-486-5p (+ others)	miR-486-5p ↑	Serum + pericardial fluid	AMI/advanced CAD	Exosomal miRNAs as stable, non-invasive PM biomarkers

## Data Availability

No new data were created or analyzed in this study.
